# Reasons People Who Use Opioids Do Not Accept or Carry No-Cost Naloxone: Qualitative Interview Study

**DOI:** 10.2196/22411

**Published:** 2020-12-23

**Authors:** Alex S Bennett, Robert Freeman, Don C Des Jarlais, Ian David Aronson

**Affiliations:** 1 Department of Social and Behavioral Sciences School of Global Public Health New York University New York, NY United States; 2 Center for Drug Use and HIV Research School of Global Public Health New York University New York, NY United States; 3 School of Social Work New York University New York, NY United States; 4 Department of Epidemiology School of Global Public Health New York University New York City, NY United States

**Keywords:** overdose, opioids, naloxone, people who use opioids, messaging, harm reduction, public health intervention

## Abstract

**Background:**

Many people use opioids and are at risk of overdose. Naloxone is an opioid antagonist used to counter the effects of opioid overdose. There is an increased availability of naloxone in New York City; however, many who use opioids decline no-cost naloxone even when offered. Others may have the medication but opt not to carry it and report that they would be reluctant to administer it if they were to witness an overdose.

**Objective:**

We aim to better understand why people who use opioids may be reluctant to accept, carry, and administer naloxone, and to inform the development of messaging content that addresses barriers to its acceptance and use.

**Methods:**

We conducted formative qualitative interviews with 20 people who use opioids who are 18 years and older in New York City. Participants were recruited via key informants and chain referral.

**Results:**

Participants cited 4 main barriers that may impede rates of naloxone acceptance, possession, and use: (1) stigma related to substance use, (2) indifference toward overdose, (3) fear of negative consequences of carrying naloxone, and (4) fear of misrecognizing the need for naloxone. Participants also offered suggestions about messaging content to tackle the identified barriers, including messages designed to normalize naloxone possession and use, encourage shared responsibility for community health, and elicit empathy for people who use drugs. Taken together, participants’ narratives hold implications for the following potential messaging content: (1) naloxone is short-acting, and withdrawal sickness does not have to be long-lasting; (2) it is critical to accurately identify an opioid-involved overdose; (3) anyone can overdose; (4) naloxone cannot do harm; and (5) the prompt administration of the medication can help ensure that someone can enjoy another day. Finally, participants suggested that messaging should also debunk myths and stereotypes about people who use drugs more generally; people who use opioids who reverse overdoses should be framed as lay public health advocates and not just “others” to be managed with stigmatizing practices and language.

**Conclusions:**

It must be made a public health priority to get naloxone to people who use opioids who are best positioned to reverse an overdose, and to increase the likelihood that they will carry naloxone and use it when needed. Developing, tailoring, and deploying messages to address stigma, indifference toward overdose, fear and trepidation about reversing an overdose, and fear of police involvement may help alleviate fears among some people who are reluctant to obtain naloxone and use the medication on someone in an overdose situation.

## Introduction

The United States is in a public health crisis involving opioid-related morbidity and mortality; overdose rates are at epidemic proportions across the country [1,2]. In response, take-home naloxone (THN) has emerged as a critical medical technology to reverse opioid-related overdose that can be used safely in community settings [3-5]. As of the late 1990s, community-based organizations—and then state and local health departments—developed overdose education and naloxone distribution (OEND) programs in response to skyrocketing rates of unintentional overdose mortality. These programs were established to equip people who use drugs, their friends, and family members with naloxone and overdose recognition, reversal, and response skills [5-8]. The size and distribution modalities of programs vary by state and city; however, these programs all typically provide no-cost naloxone to people who use opioids, along with training in how to identify an overdose, conduct rescue breathing and cardiopulmonary resuscitation (CPR), and administer naloxone [9,10]. Overdose training and naloxone are also provided to staff at many drug treatment programs and syringe service programs (SSPs), and to first responders such as law enforcement, Emergency Medical Services (EMS), and fire department personnel [11,12]. These efforts have resulted in greater access to naloxone for many individuals who might not otherwise receive the medication [13-15], and evaluations of THN programs have consistently found that these programs effectively reduce overdose mortality and lead to few adverse events [16-19].

Surveys conducted from 2013 to 2019 show that the number of SSPs with OEND programs grew from 55% to 94% in response to a dramatic rise in opioid-related overdose fatalities [20]. In pace with this expansion of naloxone distribution at SSPs, a growing body of research on naloxone access [21] and experiences emerged [22-27]. This period saw a great increase in access to OEND at SSPs and more opportunities for both people who use drugs and those who do not to obtain naloxone. However, barriers to widespread access remain, and some people who use opioids still do not carry the medication, even if they were trained and given naloxone at no cost. In a recent national survey of attitudes toward naloxone among the general population, half of the respondents endorsed that “naloxone is only necessary for people who abuse opioids,” and 51% of respondents endorsed that “having naloxone available enables more drug use among people who abuse opioids” [28]. These contested beliefs about the place and role of naloxone—who should have it and the behavioral impacts of possession and use—permeate across social networks of both people who use opioids and people who do not use drugs (or do not identify as people who use drugs) [22-27]. The belief that naloxone promotes risky drug use and that only “drug abusers” are susceptible to overdose (rather than users of prescription opioids or occasional users) diminishes enthusiasm for scaling up OEND and, thus, prioritizes other traditional supply-and-demand reduction approaches to tackling the overdose crisis.

Moreover, even among people who use opioids who are trained in OEND, there is an observed lack of acceptance of, and willingness to carry, naloxone. Among 353 Baltimore adults who reported lifetime heroin use, 90% (318/353) reported naloxone awareness, and over two-thirds (224/353, 69%) reported ever receiving take-home naloxone [29]. Of the 224 individuals who had ever received naloxone, one-third reported that they never (83/224, 37%) or rarely/sometimes (84/224, 38%) carried the medication, and only 25% (57/224) reported that they always carried the medication [29]. Commonly cited reasons for not carrying or using naloxone included fear that a person may become violent or aggressive after being revived, or that police will threaten bystanders at an overdose event, or that they had insufficient overdose training [30]. In addition to gaps in naloxone access, some people who use opioids decline the medication even when it is offered. In a small pilot study conducted in New York City, 6 of 10 participants who identified as actively using opioids accepted a THN kit when offered it free-of-charge while visiting an SSP [31]. A substantial minority (4/10), however, declined it [31]. Similarly, among 472 veterans regularly using opioids in New York City who were offered free naloxone in street-based community settings, about one-fourth (110/472) declined the free naloxone kit when offered [32].

As the above research shows, a substantial number of people who use opioids and are at risk of overdose nevertheless decline no-cost THN, opt not to carry it, and report that they would be reluctant to administer it if they were to witness an overdose. However, people who use opioids and other drugs can act as critical and effective first responders to overdose [29,33,34]; therefore, there must be minimal barriers impeding their access and use of the medication. Technology-based messaging may be one avenue to increase naloxone uptake and use. Video and text messages have been used effectively by our study team members to increase HIV/HCV testing among high-risk populations and train people who use opioids to administer naloxone [31].

To inform the development of message content addressing barriers to naloxone access, possession, and use, we recruited 20 people who use opioids in New York City from June 2019 to August 2019 to participate in in-depth qualitative interviews. The participants reported barriers they and others in their social networks have encountered in the acceptance, carrying, and use of naloxone, and they offered suggestions for overcoming these identified barriers.

## Methods

People who use opioids in New York City were recruited via chain referral and key informants affiliated with several SSPs located in the Bronx and Manhattan. Interviews were conducted by 2 experienced qualitative researchers in semiprivate settings in public parks and public spaces from August 2019 to October 2019. Interviewers traveled to the communities where the participants lived to conduct the interviews, representing 4 of New York City’s 5 boroughs. The semistructured interviews explored why people decline naloxone kits when offered, why people might be reluctant to use naloxone to reverse an overdose, and what types of messaging content could increase uptake, possession, and the likelihood of using naloxone to reverse an overdose. Participants were asked about their own experiences obtaining, carrying, and using naloxone, and they were asked about the experiences of others. Verbal consent was obtained and interviews lasted approximately 30 minutes, for which participants received $20 in cash. All interviews were digitally audio-recorded and transcribed for analysis. The institutional review board of the authors’ home institution approved all consent documents, procedures, and the interview guide.

Interviews were conducted until thematic saturation was reached (N=20). A combination of a priori and emergent code categories were used by 2 of the authors to analyze the interviews. A deductive approach to exploring the a priori topic of naloxone possession was combined with an inductive regard for the emergent themes suggested by the participants. Thereby, the project used an iterative process of reading, coding, and discussing transcripts to achieve a flexible approach to illuminating some of the forces and processes that underlie decisions about obtaining, possessing, and using naloxone [35]. The interviewers jointly coded each transcript using the Dedoose software platform (version 4.12; SocioCultural Research Consultants) and frequently met to discuss emerging themes.

## Results

### Participant Characteristics

Interviews were conducted with 20 people who use opioids. Participants were predominately male (12/20, 60%), the mean age was 37 (range 23-55) years, and the majority (16/20, 80%) injected their opioids. Of the 20 participants, 75% (15/20) identified as White, 20% (4/20) identified as Black, 10% (2/20) identified as White Hispanic, and 5% (1/20) identified as Asian. All participants had been trained in naloxone use and overdose reversal; 6 of the 20 participants were not carrying naloxone at the time of the interview.

### Stigma Related to Substance Use

The majority of participants cited drug-related—and particularly, opioid-related—stigma as one of the primary reasons people decline to carry or administer naloxone, even if they had previously accepted a naloxone kit and related training. Participants suggested that accepting or carrying naloxone would likely cause concerns for individuals who are particularly sensitive to being perceived as an illicit substance user or an addict. Several participants specifically cited the blue bag in which no-cost naloxone is distributed in New York as being nearly synonymous with illicit substance use and accompanying stigmas.

I know that some people are afraid . . . of (other) people discovering their naloxone, and that will out them as an opioid user, and I do think that those little blue bags are very recognizable. So maybe just getting rid of the blue bag would help. . . .You know, people are much less likely to recognize what it is if they don't see that, right?

36-year-old White, non-Hispanic woman

Other participants stressed the importance of normalizing the carrying or administering of naloxone in general.

When I do outreach (at a local SSP), it's like, some people will just be, like, “Oh, what you think we use, drugs?” . . . They're not comfortable with being identified as a user or whatever. I try my best to, kind of, change that perception because it's like, anybody should be able to carry Narcan. Like, it's usually not the person that's falling out that's Narcanning themselves. . . . I try to address it with basically just saying, you know . . . there's a lot of overdoses in the area, and we're giving this out to give you the opportunity to save a person's life.

27-year-old White, non-Hispanic woman

Even interviewees who were not carrying naloxone at the time of the interview (citing various reasons for not carrying it that particular day) recommended a number of steps they felt should be taken to overcome the stigma associated with carrying naloxone. These ranged from opting not to carry naloxone in the blue bag to delinking opioid use from naloxone and normalizing it in general.

I'm interested in seeing naloxone normalized. I just try to make them feel more comfortable, like (when I give people naloxone and tell them) “you guys don't have to carry it in a blue pouch if you're embarrassed. You can carry it in your pocket, you know.”

33-year-old White, Hispanic woman

I would emphasize that, you know, that there's no reason in this day and age for anybody to not carry naloxone, and everybody should know this and be carrying naloxone. It doesn't mean anything other than you're trying to, you're willing to save people's lives, or give somebody else your naloxone so they can save it. . . .The message needs to get out that, I think, the naloxone is ubiquitous. You know, just because somebody has it doesn't mean they're a drug user. I mean, there's that double-edged thing of, like, well, we don't want people to be stigmatized, but at the same time, maybe people do want, you know (to identify as a drug user). So how do, how do you find ways to talk about this shit from all the different angles? How do you have the nonstigmatizing, nonjudgmental discussion?

46-year-old White, non-Hispanic man

In nearly all of the interviews, individuals suggested that in addition to humanizing substance users in general, one particularly effective strategy for combatting substance use–related stigma surrounding naloxone might be to develop messaging and practices that actively seek to delink naloxone with active, illicit opioid use.

### Indifference Toward Overdose

Many participants also speculated that community members, referring to both people who do not use drugs and those who do, might decline to carry or administer naloxone because the lives of people who use illicit substances (and particularly opioids) are often devalued. Therefore, following this line of reasoning, some individuals simply lack the care and concern to become involved in a potential overdose situation or seek out OEND training in the first place.

Some people are just selfish and self-centered, you know? And they, and they, you know, if it ain't doesn't affect them or somebody they know, they don't give a damn.

52-year-old Black, non-Hispanic man

Based on this, participants suggested that future interventions intended to encourage individuals to carry or administer naloxone include content designed to elicit empathy and foster a shared sense of responsibility for preventing overdose-related fatalities. The point that participants thought an intervention’s messaging should make is perhaps best summed up in the simple message suggested by one participant: “You could save a life or two, you know?” [53-year-old South Asian man]. One of the strategies frequently recommended to increase community empathy was sharing personal stories from individuals who have reversed life-threatening opioid-related overdoses.

I feel good because . . . I can save a life, so that means something to me, you know? I would want somebody to do that for me, so, like, you know, there should be more people that, who want to do this ‘cause . . . it's not like you Superwoman or nothing, but it's somebody that is still gonna be around that you can see, that you can be like, “Wow, I saved that person.”

36-year-old White, non-Hispanic woman

Participants also noted that it was helpful to remind people that even individuals with whom someone is close to might be using opioids in a way that is not always apparent to others.

So, this would be, all right, say if a family member, a close family member of yours or very close friend, is dealing with this same situation, you wouldn't know, but you would like to know . . . you would like to know if they were doing it (using drugs) so I can have this with me, just in case you are doing it, I can save your life as well. For me and many others.

23-year-old Black, non-Hispanic man

Participants frequently suggested that one possibly effective strategy might be to emphasize that anyone could know someone who is at risk for a potentially fatal opioid-related overdose.

Because sometimes people don't know about something and they don't care. But once you sit down and you speak with somebody that knows what they're talking about, it opens up their mind, you know? It opens up their eyes, too. A relative could be overdosed, you know?

53-year-old South Asian man

I’ve explained that it's, you know, a really good thing to do, to give people another chance at life and stuff, you know? And that you don't have to be using drugs to carry naloxone, and if anything, I ask people to think about maybe some other people that they might know in their own life might be using drugs . . . there's a lot of overdoses in the area.

42-year-old White, non-Hispanic man

### Fear of Negative Consequences of Carrying Naloxone

Several participants expressed concerns about potential legal problems related to administering naloxone or being associated with an opioid-related overdose. Moreover, participants noted that law enforcement may mischaracterize naloxone possession as evidence that a person on parole is using drugs or associating with drug users, and that people in homeless shelters could be forcibly removed if they are found with naloxone. To address these issues, participants recommended that interventions include content designed to clarify existing Good Samaritan legislation to help people who use opioids understand their rights and address related anxieties.

I know there's this thing now (Good Samaritan Laws), and you can't get arrested or something like that. 'Cause I know a lot of people get scared with that. And they don't call 911. . . . You want to save the person. You don't want . . . a death under your belt.

31-year-old White, Hispanic woman

For many participants, this was especially important considering the likelihood that medical professionals might not reach an individual experiencing an overdose until it results in an overdose-related fatality.

Once you learn how to use it, you shouldn't be afraid to use it because you're not gonna get charged if someone . . . let's say someone's OD'ing and you come to their aid until the EMTs get there, you might have saved someone's life right there, you know what I mean?

53-year-old South Asian man

### Fears of Misrecognizing the Need for Naloxone

Many participants also expressed concern that they or others might not be able to accurately recognize an opioid overdose and, as a result, could administer naloxone to an individual who is otherwise intoxicated (eg, unconscious after using alcohol or benzodiazepines) or who is simply homeless and sleeping in public. Importantly, these concerns strongly discouraged people in our sample who reported they would otherwise administer, or at least consistently carry, naloxone.

If you're on the train and someone's really, like, nodding out, do you want to bother them and ruin their high? . . . you don't really know . . . are they drinking? Extremely drunk? And then you don't know, like, what kind of reaction you're going to get from people, ‘cause there's a certain degree of, like, mental health (problems).

55-year-old White, non-Hispanic man

Indeed, several participants suggested that misrecognizing an overdose could lead to serious negative consequences. Participants expressed that in addition to concerns regarding when naloxone use is actually warranted, there is always the possibility that a person given a dose of naloxone will immediately experience opioid withdrawal and become angry or even violent.

They don't want that naloxone to come in there and take that opiate out because it's going to make them sick, and then they don't know where their next dollar is coming from. And then someone comes along because, like, you're nodding out a little too hard or you seem like you're discombobulated, to the point where they're, like, almost dead. But if you try to get them naloxone, they'll, they'll fight you to the death not to, not to give them that, um, you know, injection or the nasal spray, because they don't want to lose that high.

41-year-old White, non-Hispanic man

Potentially negative financial consequences for someone who has had naloxone administered was also a frequently noted concern.

So, being as an addict, who wanna be sick? You know what I mean? When you gotta have, find money to buy more. You know what I'm saying? It's not like people, it's not like you're gonna give it to me. I'm sick and I'm broke. You know what I mean? I, I, I gotta find ways to get more.

54-year-old Black, non-Hispanic man

Participants underscored the role of experience in identifying, responding, and communicating safety through messaging about overdose. Participants discussed several popular myths related to reversing an overdose. In several instances, myths did, in fact, correspond to an opioid overdose reversal. However, when myths correspond to someone’s experience managing someone else’s overdose (eg, the person awakens, or regains consciousness, or becomes relatively alert), this can perpetuate myths that spread through peoples’ social networks. For example, a slap, or a yell, or just a lift up may be all that is needed to prevent an overdose from becoming more serious and requiring naloxone. Participants were aware of the myths about overdose reversal and commented on the misinformation in their communities, including potentially dangerous and not scientifically proven overdose reversal methods such as hitting a person or injecting them with cocaine or milk.

I was revived by my roommate . . . I don't know, awakened by, um, his slapping me and telling me that I was blue. But it didn't require naloxone to revive me. And he had no drug-using experience . . . to try and bring the person back, right? Which was like a lot of the folklore around, you know: throw somebody in an ice bath, beat the bottom of their feet with sticks. All these, you know, kinds of techniques that just got around on the street.

39-year-old White, non-Hispanic woman

Participants also expressed doubts regarding the severity of potentially negative physical and emotional responses to administered naloxone, and the level of certainty that an individual will respond negatively at all.

Like, just how much . . . it is sort of the myth that's out there, that you're gonna make someone very sick. I can't pinpoint . . . it's just out there, it has always been out there. People sort of take it as a given. I think it's important to know that it only lasts 90 minutes. That's important to know, too, so that if you do use it, it's not like someone's gonna necessarily be in massive withdrawal for 12 hours.

27-year-old White, non-Hispanic woman

## Discussion

### Principal Findings

Participants cited 4 main barriers that may impede rates of naloxone acceptance, possession, and use: (1) stigma related to substance use, (2) indifference toward overdose, (3) fear of the negative consequences of carrying naloxone, and (4) fear of misrecognizing the need for naloxone. Some of these barriers have been identified by other researchers, including the fear of precipitating withdrawal sickness, stigma, and the fear of arrest [12,24,36,37]. Our study participants cited stigma toward drug users and concerns of being outed as a drug user as influencing naloxone-related practices, including decisions about whether or not to carry or prominently display naloxone when carrying it. Relatedly, participants cited the public’s general indifference toward overdose and a lack of altruism as a barrier to naloxone access and use. In part, this may be associated with a perception on the part of people who use drugs of “acceptable” or “unacceptable” drug-use behaviors. For example, Bowles et al [38] found that people who frequently overdosed were often shunned by their drug-using social networks; they were considered a liability that placed others who used drugs at risk. Here, ongoing criminalization has created an atmosphere so toxic for people who use drugs that the only means of group safety and preservation is to distance from riskier drug users. However, this practice exacerbates the risk for those who are perhaps in need of the most support. It is clear that we need messaging that emphasizes naloxone as a medical technology promoting community health needs to reach community members at large. At the same time, tailored messaging is needed for people who use drugs who may themselves judge, avoid, and distance themselves from others who use drugs.

The barriers voiced by participants may not be immediately addressed simply through messaging, as many of the harms perpetrated by our dominant supply-reduction policy approach evolved over decades. For instance, stigma directed toward people who use drugs in a US context has deep historical roots [39,40]. However, what we learn from people who use opioids through this research and other efforts can help us develop tailored messaging content to overcome these barriers. Normalizing naloxone could have life-saving benefits, and destigmatizing people who use drugs could benefit their health in a broad range of communities. These messages may help move people incrementally toward regularly carrying naloxone, even if they are highly reluctant to do so initially. Many participants expressed pride when showing off their naloxone kits, strapped to belts, in bags, or on chains around necks, which we interpret as a testament to the potential for naloxone possession to be a marker of community pride and compassion for others. As Wagner et al [26] point out, reversing or witnessing an overdose can be a traumatic, cathartic, humbling, or empowering experience. Accordingly, efforts to normalize naloxone could complement participants’ messaging recommendations to emphasize shared responsibility for community health and elicit empathy for people who use drugs. Messaging could also debunk myths and stereotypes about people who use drugs more generally and help frame people who use opioids and reverse overdoses as lay public health advocates, and not just “others” to be managed with stigmatizing practices and language.

Of particular concern is the participants’ expressed trepidations about potentially misrecognizing an overdose, inadvertently precipitating withdrawal, and creating an undue financial burden on some people who use opioids, which are fears that have also been found in other research [30]. One approach to overcoming these barriers could be OEND booster messaging, deployed electronically and virtually, about protections when 911 is called, and how to identify an overdose. These messages could be coupled with other health promotion and risk-reduction messaging targeting people who use drugs. Additional resources could be allocated to community-based programs to develop and broadly distribute print and web-based messaging about best overdose-response practices and resources. Standard OEND trainings provide information on how to distinguish an overdose from a “nod” by encouraging bystanders to call out to the person if an overdose is suspected, rub the sternum or pinch an earlobe to see if there is a response, or check for breathing before administering naloxone. This information presented in OEND training could be delivered to people who use opioids on an ongoing basis, with messaging covering each point in the standard OEND curriculum. Both community-based and virtual refresher trainings could help accomplish this. Taken together, participants’ narratives hold implications for the following potential messaging content: (1) naloxone is short-acting, and withdrawal sickness does not have to be long-lasting; (2) it is critical to accurately identify an opioid-involved overdose; (3) anyone can overdose; (4) naloxone is safe to use; and (5) the prompt administration of the medication can help ensure that someone can enjoy another day.

As we grapple with the novel coronavirus, we need to remain especially vigilant. Rates of overdose continue to be high. The long-term impacts of the virus are as yet unknown, and social distancing and isolation are placing more people who use drugs at risk for a potentially fatal overdose [41-43]. Thus, compounded by the state of the COVID-19 pandemic, getting naloxone to people who use opioids is vital.

### Limitations

The findings may be unique to the population of people who use opioids in New York City, where there is robust naloxone distribution. Our findings may or may not generalize to other cities, where the overdose rate and degree of stigmatization may be different and Good Samaritan legislation may or may not be actively followed. Finally, given the nature of qualitative research, which involves small samples and nonprobabilistic sampling methods, the findings are not intended to be generalized to the broader population, suggesting the need for additional cross-sectional, longitudinal, and comparative investigations.

### Conclusions

Getting naloxone to those who are best positioned to reverse an overdose, and increasing the likelihood they will use it when needed, should continue to be a public health priority. However, the barriers identified by participants regarding naloxone access, possession, and use are considerable. Our study found a common reluctance to administer naloxone because of stigma, apathy, concerns about precipitating withdrawal, misrecognizing a good “high” as an overdose, and fears of police. These underscore the importance of distributing this proven, life-saving medication and of creating positive, acceptable messaging to ensure people use it when it is needed most.

## Abbreviations

OEND: overdose education and naloxone distribution programs
SSP: syringe service program
THN: take-home naloxone
